# Supplementary material for: CRISPR/Cas9-Mediated genomic knock out of tyrosine hydroxylase and yellow genes in cricket Gryllus bimaculatus
Source: PLoS One. 2023 Apr 10;18(4):e0284124. doi: 10.1371/journal.pone.0284124 (PMC10085040; doi:10.1371/journal.pone.0284124)
Supplement: S1 Table — (DOCX) [file pone.0284124.s001.docx]

**Supplementary Table1|** Species for the *TH* gene used in this study with GenBank accession numbers.

| Species | Accession |
| --- | --- |
| *Apis mellifera* | **NP.001011633** |
| *Apis florea* | **XP.003692921** |
| *Apis dorsata* | **XP.006619005** |
| *Bombus terrestris* | **XP.012166062** |
| *Osmia lignaria* | **XP.034181574** |
| *Polyrhachis dives* | **ACZ06878** |
| *Solenopsis invicta* | **XP.025992973** |
| *Acromyrmex echinatior* | **XP.011052688** |
| *Nasonia vitripennis* | **XP.016836527** |
| *Drosophila melanogaster* | **CAA53802** |
| *Bactrocera dorsalis* | **AVP73886** |
| *Aedes aegypti* | **XP.021696291** |
| *Anopheles sinensis* | **AMZ03511** |
| *Tribolium castaneum* | **NP.001092299** |
| *Tenebrio molitor* | **ACU77882** |
| *Bombyx mori* | **NP.001138794** |
| *Manduca sexta* | **ABQ95973** |
| *Mythimna separata* | **BAF32573** |
| *Helicoverpa armigera* | **ASS36971** |
| *Rhodnius prolixus* | **ANZ03350** |
| *Nilaparvata lugens* | **QEE04288** |
| *Gryllus bimaculatus* | **BAM15632** |
| *Ixodes scapularis* | **XP.040069107** |
