# Supplementary material for: CRISPR/Cas9-Mediated genomic knock out of tyrosine hydroxylase and yellow genes in cricket Gryllus bimaculatus
Source: PLoS One. 2023 Apr 10;18(4):e0284124. doi: 10.1371/journal.pone.0284124 (PMC10085040; doi:10.1371/journal.pone.0284124)
Supplement: S2 Table — (DOCX) [file pone.0284124.s002.docx]

**Supplementary Table2|** Species for the *yellow-y* gene used in this study with GenBank accession numbers.

| Gene | Species | Accession |
| --- | --- | --- |
| *yellow-c* | ***Bombyx mori*** | **AFC87785** |
| *yellow-c* | ***Papilio xuthus*** | **BAM18241** |
| *yellow-c* | ***Tribolium castaneum*** | **ACY71056** |
| *yellow-c* | ***Drosophila melanogaster*** | **NP.001285947** |
| *yellow-f* | ***Drosophila melanogaster*** | **NP.001262516** |
| *yellow-f* | ***Tribolium castaneum*** | **ACY71059** |
| *yellow-f* | ***Bombyx mori*** | **AFC87783** |
| *yellow-y* | ***Tribolium castaneum*** | **ACY71063** |
| *yellow-y* | ***Bombyx mori*** | **NP.001037434** |
| *yellow-b* | ***Drosophila melanogaster*** | **NP.001285987** |
| *yellow-b* | ***Tribolium castaneum*** | **ACY71055** |
| *yellow-b* | ***Bombyx-mori*** | **AFC87784** |
| *yellow-h* | ***Drosophila melanogaster*** | **NP.651912** |
| *yellow-h* | ***Apis mellifera*** | **ABB81847** |
| *yellow-h* | ***Leptinotarsa decemlineata*** | **ATB56350** |
| *yellow-d* | ***Drosophila melanogaster*** | **NP.523820** |
| *yellow-d* | ***Papilio xuthus*** | **BAM18620** |
| *yellow-d* | ***Bombyx mori*** | **AFC87786** |
| *yellow-d* | ***Tribolium castaneum*** | **ACY71057** |
| *yellow-e* | ***Drosophila melanogaster*** | **NP.524344** |
| *yellow-e* | ***Leptinotarsa decemlineata*** | **ATB56348** |
| *yellow-e* | ***Papilio xuthus*** | **BAM17806** |
| *yellow-e* | ***Bombyx mori*** | **AFC87792** |
| *yellow-e* | ***Tribolium castaneum*** | **ACY71058** |
| *yellow-g* | ***Drosophila melanogaster*** | **NP.523888** |
| *yellow-g* | ***Tribolium castaneum*** | **ACY71060** |
| *yellow-g* | ***Aedes albopictus*** | **QCI03327** |
| *yellow* | ***Gryllus bimaculatus*** | **GBI.10058-RA** |
