# Supplementary material for: CRISPR/Cas9-Mediated genomic knock out of tyrosine hydroxylase and yellow genes in cricket Gryllus bimaculatus
Source: PLoS One. 2023 Apr 10;18(4):e0284124. doi: 10.1371/journal.pone.0284124 (PMC10085040; doi:10.1371/journal.pone.0284124)
Supplement: S3 Table — (DOCX) [file pone.0284124.s003.docx]

**Supplementary TABLE 3|** Survival rate and percentage of mosaicism in F_0_ crickets injected with *TH* and *yellow-y* sgRNAs.

|  | No of eggs injected | No of incubations | Hatching rate / % | No of F_0_ mutations | Mutation rate / % | Number of mutations / total / % |
| --- | --- | --- | --- | --- | --- | --- |
| 80 ng/μL *TH* | 100 | 13 | 13.00 | 9 | 69.23 | 9.00 |
| 150 ng/ml *TH* | 100 | 10 | 10.00 | 9 | 90.00 | 9.00 |
| 300 ng/ml *TH* | 100 | 8 | 8.00 | 7 | 87.50 | 7.00 |
| 500 ng/μL *TH* | 100 | 11 | 11.00 | 10 | 90.91 | 10.00 |
| 500 ng/μL *yellow-y* | 188 | 35 | 18.62 | 25 | 71.43 | 9.89 |
| Cas9 | 100 | 24 | 24.00 | 0 | 0 | 0 |
