# Supplementary figures and images for: CRISPR/Cas9-Mediated genomic knock out of tyrosine hydroxylase and yellow genes in cricket Gryllus bimaculatus
Source: PLoS One. 2023 Apr 10;18(4):e0284124. doi: 10.1371/journal.pone.0284124 (PMC10085040; doi:10.1371/journal.pone.0284124)

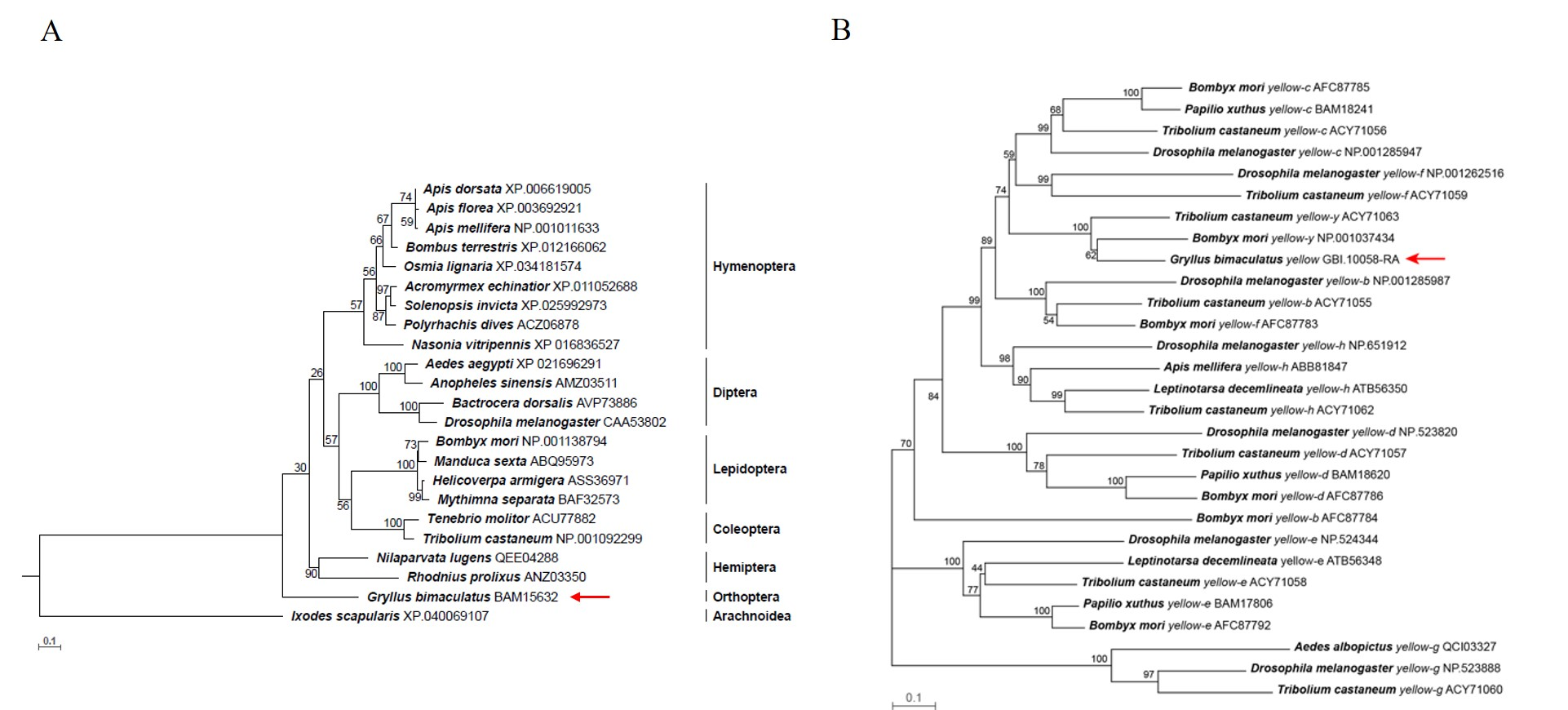

Supplement: S1 Fig — (A) Phylogenetic tree of TH protein sequences. (B) Phylogenetic tree of yellow-y amino acid sequences. (TIF) [file pone.0284124.s004.tif]

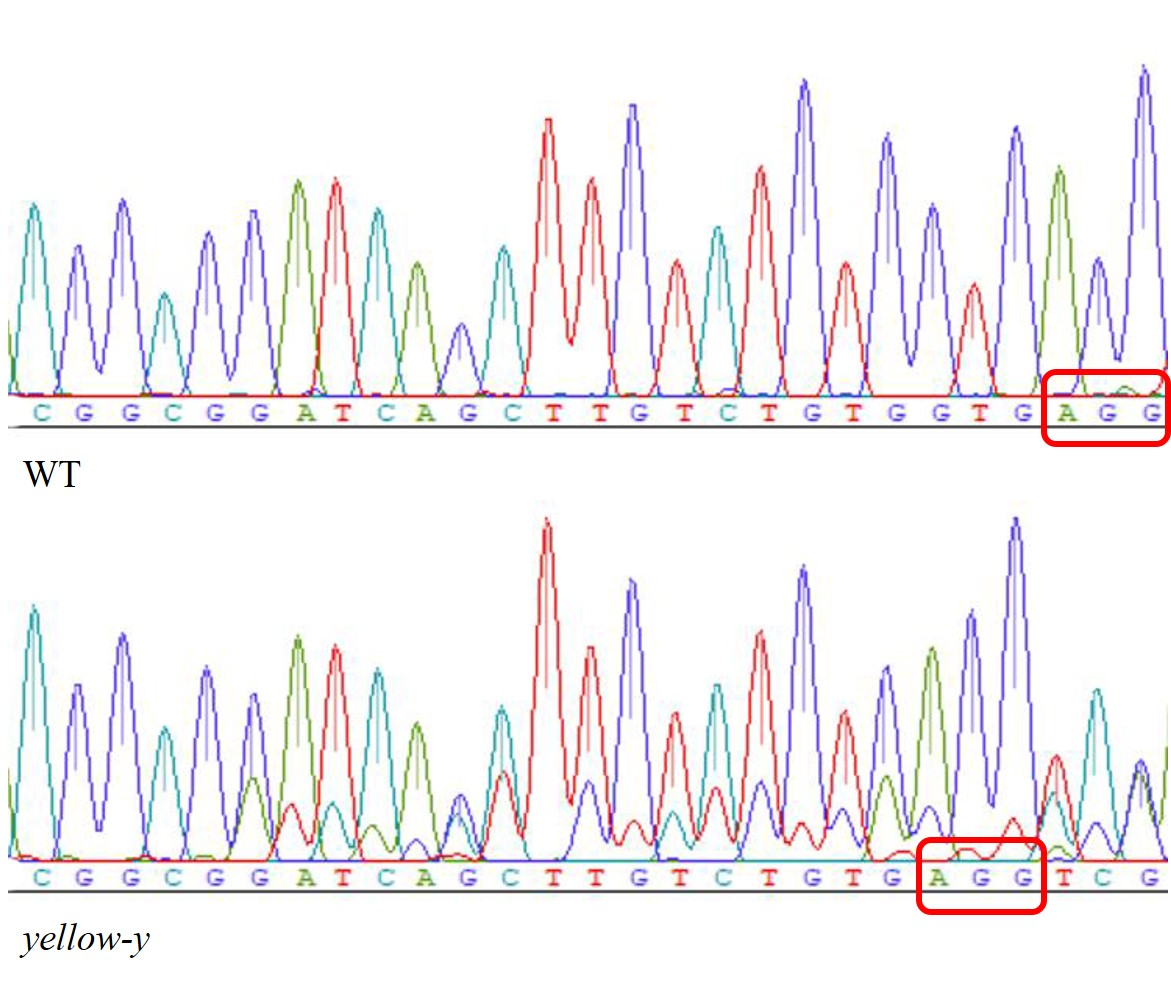

Supplement: S2 Fig — The red box refers to the PAM sequence. (TIF) [file pone.0284124.s005.tif]

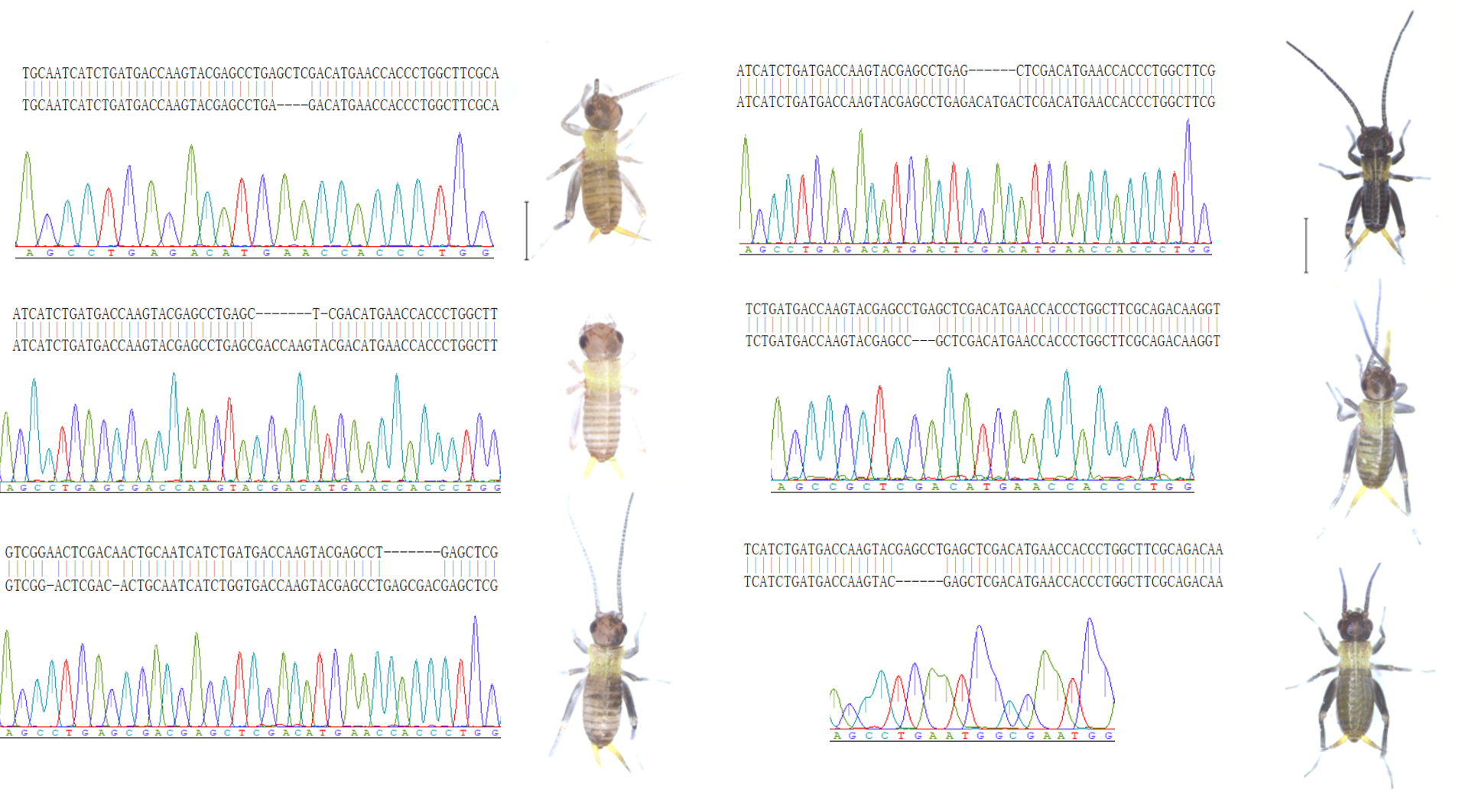

Supplement: S3 Fig — The first strand is the wild type, and the second strand is the mutant. Bar = 3mm. (TIF) [file pone.0284124.s006.tif]

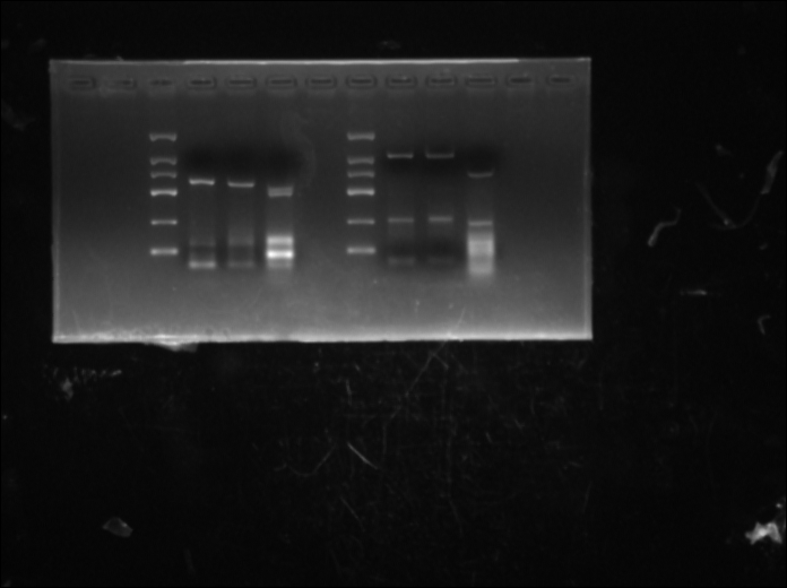

Supplement: S4 Fig — (TIF) [file pone.0284124.s007.tif]
