# Supplementary material for: CRISPR/Cas9-Mediated genomic knock out of tyrosine hydroxylase and yellow genes in cricket Gryllus bimaculatus
Source: PLoS One. 2023 Apr 10;18(4):e0284124. doi: 10.1371/journal.pone.0284124 (PMC10085040; doi:10.1371/journal.pone.0284124)
Supplement: Attachment — Submitted filename: Response to reviewers.docx [file pone.0284124.s009.docx]

**Editor:**

Thank you for submitting your manuscript to PLOS ONE. After careful consideration, we feel that it has merit but does not fully meet PLOS ONE’s publication criteria as it currently stands. Therefore, we invite you to submit a revised version of the manuscript that addresses the points raised during the review process.

Your manuscript was reviewed by three experts. They found that it is preliminary and requires a major revision(s) including additional experiments. I share their recommendations. Please revise it according to their suggestions and perform additional experiments as they suggested.

We would like to thank you first for all the suggestions of our manuscript (Manuscript Number: PONE-D-22-04580) entitled “CRISPR/Cas9-Mediated Genomic Knock out of Tyrosine Hydroxylase and Yellow genes in Cricket *Gryllus bimaculatus*”. We really appreciate your help and patience. We have seriously thought about the suggestions and provided our response to reviewers.

We added an experiment to observe the pigmentation process of *Gryllus bimaculatu* (Fig. 6), and found that the WT group completed pigmentation within 1.5h, while the mutant completed pigmentation within 24 hours. The head of the mutant is mosaic compared with the WT (Fig. 7). Dopamine concentration (Fig. 11) measurement experiments showed that knocking out the *TH* gene resulted in a significant decrease. In addition, the phylogenetic analysis of *TH* and *yellow-y* genes was added (Fig. 2). The QRT-PCR experiment (Fig. 9) and the melanin content experiment (Fig. 10) also more fully verified that the content of melanin in the mutant group was significantly reduced.

The detailed response attached below.

Please ensure that you refer to Figures 6, 7 and 8 in your text as, if accepted, production will need this reference to link the reader to the figures. Please include captions for figures 6, 7 and 8.

We thank the reviewer for these suggestions. The references and correspondence for Figures 6, 7 and 8 have been rearranged as shown in Figure 8.

We note you have included a table to which you do not refer in the text of your manuscript. Please ensure that you refer to Table 3 in your text; if accepted, production will need this reference to link the reader to the Table.

Thank you for your advice. In this article, we have modified Table 3 on page 7, line 134 to Table 1.

Please correct the following wordings:
A: Abstract, p. 2, line 28: "an efficient transient CRISPR/Cas9 system" to "an efficient transient CRISPR/Cas9 expression system"
B: Results, p. 8, line 166: "Fig 2" might be a mistake. Please cite a correct figure.
C: Results, p. 9, line 179: "Fig 3" might be a mistake. Please cite a correct figure.

We thank the reviewer for these suggestions.

A: This has been modified, see line 28 on page 2.

B: The correct image has been referenced, as shown in Figure 2.

C: The correct image has been referenced, as shown in Figure 3.

Reviewer #1:

Thank you for giving me the opportunity to review “CRISPR/Cas9-Mediated Genomic Knock out of Tyrosine Hydroxylase and Yellow genes in Cricket *Gryllus bimaculatus*” by Bai et al.
In this manuscript, the authors attempted genome editing using CRISPR/Cas9 system into the cricket, *Gryllus bimaculatus*. Then, they have successfully inducing gene knock-out mutation against *TH* and *yellow-y* genes. This genome editing method will not only benefit the cricket research community but will also be important in studies among insect species, especially for researchers who use hemimetabola insects.
However, while some potentially interesting results have been observed in this study, there are some concerns. The conclusion of this manuscript is that established the genome modification in G. bimaculatus. But it limits the novelty of this study because of already been published in other groups. Therefore, if the authors more emphasize the functions of melanin pigmentation-related genes, it will be more attractive to broad readers. This is because, from this reviewer's knowledge, findings from studies on the function of cuticular coloration in hemimetabola insects, including cricket, are not fully understood.

Major concerns:
The text is organized but there is missing information and thus the data should be incorporated in the manuscript with referred following comments.

1. The main concern to this manuscript is that the authors did not focus on expression levels of *TH* and *yellow-y* genes between wt and those mutants. Therefore, it is hard to understand the mutant phenotypes are due to null or hypomorphic mutations. It should be shown that the expression level of *TH* and *yellow-y* genes were decreased or not on the mutant by (semi-) quantitative PCR. Otherwise, at least it should be compared the phenotypes by those RNAi mutants.

Thank you very much for your suggestion, we have added this part of the experiment as shown in Figure 9.

2. Line 176 at the section of Phenotypic analysis: Please more describe details in *TH* and *yellow-y* mutants with showing dissected parts or with enlargement of the figure for the parts. In holometabola insects, the cuticular coloration pattern differs among tissues (i.e., the compound eye and the majority of body cuticles). These phenotypic differences could be observed in results (Fig. 3 and Fig. 4).

Thank you for your reminding. We have added a comparison experiment between mutant and wild-type heads, see Figure 7.

3. Is there any evidence the Gryllus *yellow-y* used in this experiment is certainly the gene classified in *yellow-y* among insects, beyond the blast search? Please take considering phylogenetic analysis using Yellow family proteins among insects including Gryllus yellow genes.

Thank you very much for your suggestion, and the *yellow* gene is a large family that we have mapped through phylogenetic analysis, see Figure 2.

4. The melanization in insect pigmentation undergoes enzymatic cascades. It is possible that mutants with weakened enzymatic activity may be slower than normal in coloration. Please show the time course changes in body coloration of the wild type and the mutant during development.

Thank you for your advice. We have added experiments to observe the process of body color changes in mutants and wild-types in Figure 6.

5. Please prepare the table having injection statistical numbers. The table should have at least numbers of Injected eggs, Hatched nymphs, Visible mosaic adults. Adding for *yellow-y*, it should have the number of F0 adults crossed and F_1_ with color mutation.

Thank you for your suggestion. We have explained the injection of *yellow-y* mutants by adding Tables 3 and 4.

Minor concerns:

6. Fig. 1 and 2, It is hard to understand where gRNA is bound in present figures in which the sequences were shown by amino acids. It should be shown gRNA location with the sequence of nucleic acids.

Fig. 1 and 2, Is there any functional domain in those genes? Please show overlaying the domains into current figures.

Thank you very much for your suggestion, and we have re-experimented and plotted the graphs validated in vitro, see Figure 3.

7. Line 105, Please describe what type of PCR enzyme was used for producing gRNA in this experiment.

Thank you for your suggestion. This has been added. See page 6, line 121.

8. Lin 219, Please use Holometabola and Hemimetabola to specify the metamorphic type.

Thank you for your suggestion. This has been modified. See page 19, line 347-348.

9. Figure 3, Please center the PAM sequence in the sequence wave. This will give information on the mutation status of the surrounding 5’ and 3’ sequences.

Thank you for your advice. We have modified the gel map and sequence orientation, see Figure 3.

Reviewer #2:

The manuscript by Yun Bai et al., entitled “CRISPR/Cas9-Mediated Genomic Knock out of Tyrosine Hydroxylase and Yellow genes in Cricket *Gryllus bimaculatus*”, addresses the methods of gene knock-out using CRISPR/Cas9 system, targeting two pigmentation genes *TH* and *yellow-y*.
The Authors obtained *TH* knock-out F0, and *yellow-y* knock-out F0 and F1, using single gRNA and Cas9 protein, and showed their edited sequences and pigmentation defect phenotypes. In *Gryllus bimaculatus*, gene knock-out and targeted gene insertion mediated by gRNA and Cas9 mRNA are established. The authors claimed that genome editing using Cas9 protein is the preferred choice in *Gryllus bimaculatus*, like in *Pyrrhocoris apterus*, based on their results. However, genome editing method using Cas9 protein in G. bimaculatus is already established (Ohde et al., 2022), and authors did not fully perform to confirm their genome editing results. Thus, the novelty of their findings is quite limited.

Major comments

In figure 1, genome structures of *TH* and *yellow-y* genes are incorrect. *TH* gene (BAM15632.1) and *yellow-y* gene (GBI_10058-RA) contain at least 11 and 4 exons, respectively, in G. bimaculatus genome.

Thank you for your suggestion. The exons of the *TH* and *yellow-y* genes of *Gryllus bimaculatus* have been modified by reviewing the transcriptome data and alignment with *Drosophila* sequences, see Figure 1.

Line 166 and Figure 2 A, B. The authors briefly describe the results of in vitro Cas9 cleavage assay, but the authors should describe their results more carefully. There are no information about the size of DNA marker. In figure 2 A, the amounts of DNA samples are different in each lane; the amounts of DNA in lanes 2 and 3 looks much larger than in lanes 3 and 4. I think Cas9 cleavage assay should be done to same amount of DNA. In figure 2B, band patterns of lanes 2-7 were faint and out-of-focus, thus, it was difficult to distinguish the cleavage pattern. The authors should perform in vitro cleavage assay using same amounts of DNA fragments and show the lengths of DNA fragments or DNA marker.

Thank you very much for your suggestion, and we have re-experimented and plotted the graphs validated in vitro, see Figure 3.

lines 173-175. The authors claimed that DNA sequences of injected group showed multiple peaks, in figure 2B and C. The authors showed WT and *TH* in complimentary, but should show normal directions. The authors need to add the explanation about the red rectangles (maybe those indicate PAM sequences?).

Thank you very much for your suggestion, and we have re-experimented and plotted the graphs validated in vitro, see Figure 3. The red rectangle represents the PAM sequence, see page 13, line 223.

About the phenotypic analysis. The authors showed *TH* and *yellow-y* knocking-out F0 nymphs in figure 3. In my knowledge, cricket exoskeleton is formed several layers and some layers accumulate melanin pigment, but other layers does not. The authors focused on the pigmentation genes functions in this paper, the authors should show the pigmentation defects in the exoskeleton by sectioning WT and injected F0 nymphs. Such experiments would provide the data related to the mosaicism of genome editing F0 nymphs using CRISPR/Cas9 system.
After melanin synthesis gene knocking-out, however, crickets showed brownish body color. The authors should show the results, or discuss, about other pigments, especially about ommochromes, which appears brownish or reddish color.
The authors claimed that most of *TH* genome editing F0 nymphs died before or during the first molting, but the authors did not mention about the reasons of their nymphal lethal phenotype. *TH* catalyses tyrosine to dopa, and dopa is not a substrate of melanin but also dopamine. I recommend that the authors should detect and compare the amount of dopamine in WT and *TH* genome editing nymphs, because dopamin is involved neuronal activeties.

Thank you for your advice. The cuticle is the outermost part of the insect body, and its physical properties, for example elasticity, thickness, strength and stiffness, are key determinants in maintaining insect locomotion, mechanical support, body shape and normal development (Wan *et al*., 2016). As the insect exoskeleton, the cuticle is also the first protective barrier to defend against multifarious environmental stresses and prevents the penetration of external compounds, including pathogens, dehydration, physical injury and insecticides (Balabanidou *et al*., 2018). However, the hard insect exoskeleton impedes further growth during molting and metamorphosis. Therefore, cuticle digestion and biosynthesis must occur periodically in insects. It is well known that the newly synthesized insect cuticle is pale and soft, and must undergo a tanning process (sclerotization and pigmentation) over a specific period to allow normal insect growth and development (Gorman and Arakane., 2010). The level of *TH* in the insect integument can influence the degree of cuticle tanning in *Manduca sexta* (Gorman *et al*., 2007). In insects, body pigments are mainly synthesized in epidermal cells through a complex cascade of biochemical reactions. Body pigments are important for insect morphology and protection insects from exogenous physical injury (Andersen, 2010).

The color of insect compound eye is determined largely by the nature of pigments (Ichiki *et al*., 2007). Xanthommatin (brown pigment) and pteridine (red pigment) are two key forms of this pigment (page 18, line 332 and page 19, line 333-341).

See Figure 11 for the dopamine content in the mutant group and the control group.

Wan C, Hao Z, Feng X. Structures, properties, and energy-storage mechanisms of the semi-lunar process cuticles in locusts. Scientific reports, 2016, 6(1): 1-13.

Balabanidou V, Grigoraki L, Vontas J. Insect cuticle: a critical determinant of insecticide resistance. Current opinion in insect science, 2018, 27: 68-74.

Gorman M J, Arakane Y. Tyrosine hydroxylase is required for cuticle sclerotization and pigmentation in *Tribolium castaneum*. Insect biochemistry and molecular biology, 2010, 40(3): 267-273.

Gorman M J, An C, Kanost M R. Characterization of tyrosine hydroxylase from *Manduca sexta*. Insect biochemistry and molecular biology, 2007, 37(12): 1327-1337.

Andersen S O. Insect cuticular sclerotization: a review. Insect biochemistry and molecular biology, 2010, 40(3): 166-178.

Ichiki R, Nakahara Y, Kainoh Y, et al. Temperature‐sensitive eye colour mutation in the parasitoid fly *Exorista japonica Townsend* (Dipt.: Tachinidae). Journal of Applied Entomology, 2007, 131(4): 289-292.

The authors showed the photos of *TH* or *yellow-y* genome editing nymphs that appear mosaicisms in figure 3, and the authors determined the specific mutation types from these genome editing nymphs and showed the sequences in figure 5. However, the authors did not specify which sequences were obtained from which CRISPANTs. In figure 5, some sequences have 3, 6, 21 bp deletion or 9 bp insertion that leads in-frame mutation, thus, mutated *TH* or *yellow-y* protein have 1, 2, 7 amino acids deletion or 3 amino acids insertion. Generally, these small in-frame deletion or insertion may not affect an enzymatic activity. In figure 5 A, *TH* genome editing nymph in the most left panel shows blackish pigment pattern, similar to the WT or Control, speculating that these blackish nymphs have in-frame mutation. The authors should carefully show the relationship between mutation patterns of *TH* and *yellow-y* genes and mutant phenotypes of pigmentation.

Thank you very much for your suggestion, and in the study of mutant phenotypes in this paper, individuals whose base pair insertion or deletion is not 3 times are selected, so these individuals all have frameshift mutations (page 10, line 165-166).

In Table 2 and Figure 5B, the authors showed phenotypes of *yellow-y* / WT F1 nymphs. The authors showed a ratio of mutant and WT as nearly 50% and 50% in both *yellow-y* male / WT female and *yellow-y* female / WT male. Is this ratios mean that genetic locus of *yellow-y* is on autosome, not on a sex chromosome? The authors should discuss about the ratio of F1 generation.

Thank you for your advice. This has been added to the discussion section, see lines 370-373 on page 20.

In figure 5 D, the authors showed DNA sequencing results of *TH* and *yellow-y* genome editing eggs and WT. Again, the authors showed the sequences of *yellow-y* in complementary, but should be shown in normal direction. In the figure legends, the authors wrote that the red rectangles in the figure are the PAM sequences. In figure 5 D, four letters CTCG in *TH* and GGTG in *yellow-y* were boxed in red rectangles, but the PAM sequences of Cas9 is three letters (NGG), not four letters. The authors should show the correct PAM sequences.

Thank you for your advice. The red boxes represent missing sequences. It has been modified on page 16, line 278.

In general, expressions of the genome-edited genes (in this case, *TH* and *yellow-y*) of CRISPANTs and WT should be determined and compared in mRNA level and protein level, by using qPCR (or northern blot) and Western blot, in addition to the determination of DNA sequencing of targeted sites.

Thank you very much for your suggestion, we have added this part of the experiment as shown in Figure 9.

As the author mentioned in Discussion section, the authors should determine whether mutations occur in off-targets or not.

Thank you for your advice. As mentioned earlier, there are many available online CRISPR design tools for predicting off-targets of particular sgRNAs (Cui *et al*., 2018). However, in our case, where information on the complete genome is unknown, the prediction of off-target effects by any of the design tool is impossible. Even with the draft of the genome available, where the manual search of off-targets (search against the occurrence of the “seed” region of the crRNA (Cho *et al*., 2014; Zhang *et al*., 2015) can be performed, we cannot exclude the possibility that some of the off-targets exist in unassembled repetitive regions (page 18, line328-331).

Cui Y, Xu J, Cheng M, et al. Review of CRISPR/Cas9 sgRNA design tools. Interdisciplinary Sciences: Computational Life Sciences, 2018, 10(2): 455-465.

Cho S W, Kim S, Kim Y, et al. Analysis of off-target effects of CRISPR/Cas-derived RNA-guided endonucleases and nickases. Genome research, 2014, 24(1): 132-141.

Zhang X H, Tee L Y, Wang X G, et al. Off-target effects in CRISPR/Cas9-mediated genome engineering. Molecular Therapy-Nucleic Acids, 2015, 4: e264.

Minor comments

line 66 (so called "insertions") should be (so called "indels").

Line 122 "The vitro" should be "The in vitro".

Line 150 "TM" in "AxyPrep TM Multisource Genomic DNA Minipres Kit" should be in superscript.

Line 269 "Lim, K.-T.J.P.o." should be "Lim, K.-T.".

Line 282 ""Noji, S.J.S." should be "Noji, S.".

Line 295 The authors names and journal name are incorrect.

Lines 351-354 The authors names are incorrect.

Line 388 and 389-390 G. bimaculatus should be in italic.

Line 388 and 390 "5nd" should be "5th".

Line 395 "8nd" should be "8th".

We thank the reviewer for these suggestions. We have revised the above problems in the article.

Reviewer #3:

In this paper, the authors examine function of *TH* and yellow genes in a model insect species, *Gryllus bimaculatus*, in the melanin pathway. They use CRISPR to target these genes and demonstrate mutant phenotypes. The results suggest that the mutagenesis using Cas9 protein rather than Cas9 mRNA is feasible for this species, and that mutagenesis for both *TH* and yellow genes causes body color chages in nymphs. The conculsion of this paper seems reasonable; however, the data presented in this study are rather preliminary and I have concerns about the quality of the data presented in the paper and the description of the results.
Overall, I feel that the paper is too preliminary to be considered for publication.

Major comments:

1) Although this paper emphasizes the success of mutagenesis using the Cas9 protein as an achievement, the use of the Cas9 protein is already a widely known method and is not novel, even if it is the first report of its use in crickets. Even leaving novelty aside, if the efficacy of Cas9 protein is to be emphasized, at least comparative data with the use of mRNA against the same target are needed.

Thank you for your advice. There are three primary sources of Cas9 delivery used for injections in genome editing experiments: (a) expression plasmid producing Cas9 mRNA and translated to protein in the host cell, (b) mRNA of Cas9, and (c) Cas9 protein (Bassett and Liu, 2014; Housden et al., 2014; Gratz et al., 2015; Kistler et al., 2015; Thurtle-Schmidt and Lo, 2018). In previous studies, further experiments were performed with a higher concentration of the Cas9 protein (500 ng/µl) in the injection mixture. Increased efficiency with the use of the Cas9 protein in the mutant generation is most probably caused by the higher stability in the host cells (Kotwica-Rolinska *et al*., 2019). And injecting the complex of sgRNA and Cas9 protein at the region of zygote formation allows Cas9 nuclease to edit genes immediately after injection (Hu *et al*., 2019). Both of Cas9 plasmid and mRNA could not function immediately (Kouranova *et al*. 2016); they need a certain time to experience a protein synthesis process in the embryo (Kouranova *et al*. 2016) and may miss the most suitable editing time (page 18, line 318-324).

Bassett A, Liu J L. CRISPR/Cas9 mediated genome engineering in Drosophila. Methods, 2014, 69(2): 128-136.

Housden B E, Lin S, Perrimon N. Cas9-Based Genome Editing in Drosophila. The Use of CRISPR/cas9, ZFNs, TALENs in Generating Site Specific Genome Alterations 546. 2014.

Gratz S J, Rubinstein C D, Harrison M M, et al. CRISPR‐Cas9 genome editing in Drosophila. Current protocols in molecular biology, 2015, 111(1): 31.2. 1-31.2. 20.

Kistler K E, Vosshall L B, Matthews B J. Genome engineering with CRISPR-Cas9 in the mosquito Aedes aegypti. Cell reports, 2015, 11(1): 51-60.

Thurtle‐Schmidt D M, Lo T W. Molecular biology at the cutting edge: a review on CRISPR/CAS9 gene editing for undergraduates. Biochemistry and molecular biology education, 2018, 46(2): 195-205.

Kotwica-Rolinska J, Chodakova L, Chvalova D, et al. CRISPR/Cas9 genome editing introduction and optimization in the non-model insect Pyrrhocoris apterus. Frontiers in physiology, 2019: 891.

Hu X F, Zhang B, Liao C H, et al. High-efficiency CRISPR/Cas9-mediated gene editing in honeybee (Apis mellifera) embryos. G3: Genes, Genomes, Genetics, 2019, 9(5): 1759-1766.

Kouranova E, Forbes K, Zhao G, et al. CRISPRs for optimal targeting: delivery of CRISPR components as DNA, RNA, and protein into cultured cells and single-cell embryos. Human gene therapy, 2016, 27(6): 464-475.

2) Result page9：It is not appropriate to argue the function of *TH* from the phenotype at F0 described in this paper. There is a lack of analytical data on the phenotype (e.g., effect on cuticle thickness). In particular, although it is reasonable to assume that the lack of dopamine synthesis does indeed affect body color, it is difficult to determine from the results obtained whether this is a systemic or localized function. Although it is stated that most *TH* knockouts are lethal, not all, and it is considered possible to obtain the next generation by reducing the amount of Cas9 introduced, etc. *TH* gene function should be considered based on the phenotype of the heterozygous or homozygous knockout.

Thank you for your advice. We have obtained more mutants by reducing the injection volume, and will follow-up by constructing pure and mutant mutants to study the function of *TH* and *yellow-y* genes and more detailed tissue localization.

3) Line 183, Fig.4: The F1 phenotype of the Yellow gene knockout is described as brown or black body color. However, the correspondence between the described phenotype and genotype is unclear in the paper. It is essential to clarify the exact correspondence with the genotype in order to infer the gene function from the phenotype of the mutant.

Thank you for your advice. All mutants for subsequent functional verification were selected from individuals whose number of mutated bases was not a multiple of 3. The sequence map of the number of missing bases presented in Figure 8 is to reflect the mutation efficiency.

4) The insect yellow gene family generally contains a large number of genes (as the authors also state in line 231 of the text). More detailed verification and description of which genes of the gene family have been knocked out is needed to reach the conclusions of this paper. Can the gene targeted by the authors be considered a true ortholog of the Drosophila yellow gene? The specificity of genome editing must also be verified, as genes of the same family are likely to have similar nucleotide sequences.

Thank you for your advice. We have performed a phylogenetic analysis of the *yellow-y* gene, see Figure 2.

Minor comments:

5) Fig. 1, 2: For each of the *TH* and Yellow proteins, the functional domains should be shown and the targets of the guide RNA should be indicated.

Thank you very much for your suggestion, and we have re-experimented and plotted the graphs validated in vitro, see Figure 3.

6) All of the individuals showing the phenotypes shown in Figs. 3 and 4 appear to have abnormal antennae. Is the effect on the antennae significant?

Thank you for your advice. Because the crickets were only slightly anaesthetized during the photo shoot, they also had a slow motion. So far, these genes have no effect on the development of crickets' antennae.

7) Method: The authors should specify the database used to identify the *TH* and yellow genes in crickets.

Thank you for your advice. In line 94-99 on page 5, database NCBI and InsectBase have been given.

8) line 225: I could not understand the meaning of this sentence.

Thank you for your advice. Lines 354-356 on page 20 have been revised.

9) line 230-231: In the results section, there appears to be no description of the body defect due to *TH* gene knockout mentioned here. Also, what specific data is the statement that it is essential for "growth and development" based on? At least lethality does not necessarily mean that it is involved in growth and development.

Thank you for your advice. This is explained on page 20, line 361-362.

10) Table1: This table is difficult to comprehend. What do the numbers in "gonadal mosaics" mean? Does it mean the efficiency of introducing mutations into the germplasm? It is also unclear what the numbers in Number of mutations mean. An explanation of how these numbers were calculated is needed.

We thank the reviewer for these suggestions. "Gonadal Mosaics" has been modified in Table 2. In addition, the data in the table are completed by statistical method of variance calculation for three repeated experimental data.
