# Supplementary material for: CRISPR/Cas9-Mediated genomic knock out of tyrosine hydroxylase and yellow genes in cricket Gryllus bimaculatus
Source: PLoS One. 2023 Apr 10;18(4):e0284124. doi: 10.1371/journal.pone.0284124 (PMC10085040; doi:10.1371/journal.pone.0284124)
Supplement: Attachment — Submitted filename: Response to reviewers.docx [file pone.0284124.s010.docx]

**Dear** **Editor,**

We would like to thank you first for all the suggestions of our manuscript (Manuscript Number: PONE-D-22-04580) entitled “CRISPR/Cas9-Mediated Genomic Knock out of Tyrosine Hydroxylase and Yellow genes in Cricket *Gryllus bimaculatus*”. We really appreciate your help and patience. We have seriously thought about the suggestions and provided our response to reviewers.

In Fig. 1, the structure of *TH* and *yellow-y* genes is analyzed in more details, for example, coding region and UTRs structure are supplemented. We show the deformity of leg and wing by *TH* gene knocking out of *Gryllus bimaculatus* by adding Fig. 5. We re-analyzed the qPCR data of *TH*, *yellow-y* and *Ddc* genes and normalized them, as shown in Fig. 10 and 12. We add references and related discussions on the interaction between *TH* and *Ddc* genes. *TH* genes show different spatio-temporal time and tissue expression patterns in different species, which may be closely related to the upstream and downstream regulatory elements (Gorman et al., 2007; Yu et al., 2011). In addition, we add supplementary materials (Supplementary Fig. 2) to explain the phenotypic and functional differences between the gene changes of multiples and non-multiples of three base-pairs. We illustrate the specific mutation types of *TH* and *yellow-y* genes in more details in the supplementary material Fig. 1 and 3.

Gorman M J, An C, Kanost M R. Characterization of tyrosine hydroxylase from *Manduca sexta*. Insect biochemistry and molecular biology, 2007, 37: 1327-1337.

Yu H S, Shen Y H, Yuan G X, et al. Evidence of selection at melanin synthesis pathway loci during silkworm domestication. Molecular biology and evolution, 2011, 28: 1785-1799.

Reviewer #1:

The authors have addressed most of the criticisms and performed several suggested experiments to address critical points. Although the authors responded well to the reviewers' comments several minor changes are still required.

I don’t know whether the journal accepted the style, but it makes it hard to read the figure legends embedded in the result section. The figure legends should be in a different section.

Thank you for your suggestion. We modified it.

In Figure 1, please include UTRs and CDS to understand gene structures.

We have modified it, as shown in new Fig. 1.

Line 216 and Figure 3. The author performed in vitro cleavage assay to evaluate gRNA efficiency. Could you please specify the size (length) of the target amplicon? Then also, please show the size of the cleaved amplicon.

Thank you for your advice. The target strip can be cut into two segments of different sizes. The original length of *TH* gene is 545bp, which is digested into 464bp. The original length of *yellow-y* gene is 1058bp and is digested into 826bp. We added these details in revision, please see lines 204 to 207 on page 12.

Line 93 and Figure 1. Please organize the accession number of protein sequences in insects that are used in the phylogenetic analysis in a supplemental text.

We revised it, please see supplementary material Table 1 and Table 2.

Line 181. Please describe what tissues were used, sample number, and biological replicates leading to the statistical changes by the qPCR.

Thank you for your advice. qPCR was performed to analyze the expression levels of *TH* and *yellow-y* genes with three 1st-instar nymphs of mutant and WT. All experiments were performed in three biological replicates. We have added these details in the text, see page 10, lines 168 to 173.

Reviewer #2:

The authors added new data and figures suggested by review comments. But the manuscript is still not well written especially in the Results section, and authors did not discuss about new data. The figures, graphs, and tables appear to be improperly prepared. Therefore, the authors need to rework the figures and graphs appropriately. The author claimed that main focus of this manuscript is establishment of genome editing strategy by using Cas9 protein, however, the strategy is already established from other laboratory (https://doi.org/10.1038/s41467-022-28624-x), as I mentioned in my previous review comments, although the authors did not cite this reference and discuss about the strategy. Therefore, the authors should shift the focus from establishment of genome editing strategy to molecular functions of pigmentation process.

We thank the reviewer for these very meaningful suggestions. When we wrote this manuscript, this research had not yet been published. In new revision, we refer to the new article on the construction of stable mutant strains of *Gryllus bimaculatus* by injecting Cas9 protein, see lines 265-267 on page 15 and see lines 304-305 on page 17. Following the advice, we will further study the gene function of body color-related genes and the underlying molecular mechanisms by achieving homozygous mutant strains.

Major comments:

1. About abbreviation. The author uses abbreviations such as "WT," "CRISPR," "*TH*," "mRNA," "SE," and "Gryllus." Generally, when an abbreviation appears for the first time, it is given in full spelling, and the next time it appears, all abbreviations are given in abbreviated form. In this manuscript, the full spelling and abbreviations appear several times, making it difficult to read.

Thank you for your advice. All abbreviations have been checked and revised.

2. Line 210-212

The authors roughly wrote the results of in vitro verification, but the authors should show the results in detail. Original lengths of *TH* and *yellow-y* PCR products and predicted lengths after in vitro verification should be shown. Since there are no information on the sizes of PCR products and digested segments, it is impossible to determine if the experiments were successful or not.

Thank you for your advice. The target strip can be cut into two segments of different sizes. The original length of *TH* gene is 545bp, which is digested into 464bp. The original length of *yellow-y* gene is 1058bp and is digested into 826bp. We added these details in revision, please see lines 204 to 207 on page 12.

3. Line 213 and others

About figure legends. Please add a line break after the figure description, instead of continuing it with the main text.

Thanks. We have modified it.

4. Line 220-223 and Table 2

The authors wrote the percentages of mutant rate in main text and Table 2, but the values are different between them. I think the authors showed rounded values in Table 2, but the values should be in consistent.

In some case, the values are completely different between main text and table. For example, rounded value of "7.4%" in the main text is not "8%" in Table 2 for 300 ng/uL case. I am concerned about whether there is a data mix-up.

Thank you very much for your suggestion. The data in previous table was obtained by analysis of variance anova, while the data in the text was processed by means. This has now been carefully checked and changed to a uniform format. We corrected it in revision, see lines 208 to 214 on page 12.

5. Line 224-225

The authors claimed that the mutant showed double peaks or random peaks at the target site. But in *yellow-y* mutant, only few double peaks are observed far from the PAM sequences. The authors should add the explanation about the direct sequence data of figure 3B and D.

In addition, PAM sequences shown by red boxes in figure 3D is different from the PAM sequences shown by red texts in figure 8B, so it is impossible to determine which is the actual PAM sequence.

Thank you very much for your suggestions. Mutations in the gene were random at the target, and sequencing results close to PAM sequence mutations were added in the supplementary material Fig. 1. We have made further supplementary explanations to Fig. 3B and D, as shown in Supplementary Fig. 1. In addition, the annotations have been modified in Fig. 8B and D, and the annotation format of PAM has been unified. The red box represents the PAM sequence and the green box represents the missing base sequence.

6. Line 243-244, Line 254, and Figure 7

The authors wrote "The mutant heads of *TH* and *yellow-y* genes are mosaic-shaped (Fig7)" in line 243-244. In line 254, The authors wrote "(B) *TH* mutant HEAD pigment patterns. (C) The HEADS of *yellow-y* mutants are mosaic.", but in Figure 7, the authors showed TWO *TH* mutants heads and ONE *yellow-y* mutant head. The numbers of heads in Figure 7 and figure legend are different, so I could not understand which photo is *TH* mutant(s) and *yellow-y* mutant(s).

The second head In figure 7 seems bright and mosaic pigment patterns, but the third and fourth heads seems darker and uniformed pigment pattern. Thus, I think that the second head is *TH* mutant and the third and fourth head are *yellow-y* mutant, but it is still unclear why *yellow-y* mutant heads showed uniformed pigment pattern. The authors should present the generation of these mutant. I think *TH* mutant is F0 generation but *yellow-y* mutants could be F1. I am concerned that there may be a data mix-up.

Thank you for your advice. In Fig. 8, two *TH* mutant heads and one *yellow-y* mutant head are shown. The mutant heads of *TH* and *yellow-y* genes are mosaic-shaped, with the *TH* mutant having a light brown and black mosaic head, and the *yellow-y* mutant having a dark brown and black mosaic head. We have revised the description in the figure note, see lines 480 to 482 on page 33. The "head" in Fig. 8 is not plural, which caused misunderstood.

7. Lines 266-267, 270-271 and figure 8 The authors describe the results of DNA sequencing after TA cloning of *TH* and *yellow-y* mutants. The authors showed thirteen sequences of *TH* F0 and three and three sequences of *yellow-y* F0 and F1 in figure 8A and 8B, but explanation about sequencing results are too rough in line 270-271. Are there differences in pigment patterns between indels? For example, -21 bp, -6 bp, -3 bp, and +9 bp will not cause frame-shift mutation, thus mutated tyrosine hydroxylase would work and mutants containing these indels may show normal pigment pattern. Mutant nymphs containing other indels may cause frame-shift mutation and may produce non-functional enzyme, so these mutant nymphs may show defects in pigmentation. The authors should describe their experimental results in more detail.

In figure 8C and 8D, the authors showed wave data of *TH* mutant and *yellow-y* mutant in F1 generation. Both mutated sequences showed 4 bp deletion, but the authors should show the reason why they showed only these sequences.

The directions of nucleotide sequences and wave data shown in figure 8A, 8C and figure 3B are opposite. In addition, for *TH* gene case, the PAM sequences in figure 8A (the authors showed it in red) is different from the PAM sequences in figure 3B. For the *yellow-y* case, PAM sequences of figure 8B, 8D and figure 3D are different. Which is the correct PAM sequences? Where is the target site? The authors should correct figure 8 and figure 3, showing correct target sequences and PAM sequences.

Thank you very much for your suggestions.

In revision, we added the correlation between base pair deletion being a multiple of three or not a multiple of three and phenotype, and we also list some phenotypes, as shown in supplementary Fig. 2. The phenotype is clear when the number of missing bases is not a multiple of three.

Some representative results are selected for display in Fig. 8. We show more mutation types, such as missing 7bp and adding 1bp, in the supplementary materials Fig. 2 and Fig. 3.

The annotations have been modified in Fig. 8B and D, and the annotation format of PAM has been unified.

8. Line 277-278

The authors showed down-regulation of *TH* and *yellow-y* transcripts in these mutants. The authors designed target sites of sgRNA in coding region, not in regulatory elements of these genes, so why transcripts were decreased? I have often experienced such down-regulation of target gene's transcripts. The authors need to discuss about the down-regulation of the target genes citing appropriate references.

It was reported that the dietary *TH* double-stranded RNA led to reduced target gene transcript levels and larval feeding levels and caused larval mortality of diamondback moth (Ellango et al., 2018). In addition, in the study of *TH* gene function mediated by CRISPR/Cas9 gene editing technology in *Agrotis ipsilon*, it was also found that the expression level of *TH* gene in the mutants was also significantly decreased. Compared to RNA interference technology inhibiting gene expression at the transcription level, the CRISPR/Cas9 system works at genomic level, which is more destructive to gene function. *TH* genes show different spatio-temporal expression patterns in different species, which may be closely related to the upstream and downstream regulatory elements (Gorman et al., 2007; Yu et al., 2011). In *Tenebrio molitor*, the *Ddc* gene is located downstream of the *TH* gene (Mun et al., 2020). This part has been added in the article, please see page 18, lines 318-328.

Ellango R, Asokan R, Chandra G S, et al. Tyrosine hydroxylase, a potential target for the RNAi-mediated management of diamondback moth (Lepidoptera: Plutellidae). Florida Entomologist, 2018, 101: 1-5.

Gorman M J, An C, Kanost M R. Characterization of tyrosine hydroxylase from *Manduca sexta*. Insect biochemistry and molecular biology, 2007, 37: 1327-1337.

Yu H S, Shen Y H, Yuan G X, et al. Evidence of selection at melanin synthesis pathway loci during silkworm domestication. Molecular biology and evolution, 2011, 28: 1785-1799.

Mun S, Noh M Y, Kramer K J, et al. Gene functions in adult cuticle pigmentation of the yellow mealworm, *Tenebrio molitor*. Insect biochemistry and molecular biology, 2020, 117: 103291.

9. Line 279-283

About figure 9. The authors showed relative expression levels of *TH* and *yellow-y*, and they set the mean of the relative expressions in WT to 1, but in figure 9, bars of *TH* and *yellow-y* of WT are slightly different from 1.0 of vertical axis. Especially in Figure 9A, gray bar of *TH* relative expression appears to be stretched vertically. The authors must rework the graph. The authors added asterisks to *TH* and *yellow-y* relative expressions, but there is no information about the numbers of asterisks. The authors should show the P values of student's t-test.

Thank you very much for your suggestion. We re-analyzed the qPCR data of *TH*, *yellow-y* and *Ddc* genes and normalized them, as shown in Fig. 10. Four stars represents a P value less than 0.0001.

10. Line 285

In Table 4, the authors showed the ratio of Mutant and WT as numbers of offspring and percentage. For *yellow-y* male x WT female case, sum of Mutant (58.5%) and WT (46.8%) percentages exceeds 100%. In general, sum of percentage never excess 100%. Are individuals with mosaic pigment patterns counted as both mutant and wild type? The authors need to explain why.

Thank you for your advice. This data is due to a calculation error and has been corrected in revision. The correct results should be 53.19% and 46.81%.

11. Line 290-291

The authors showed down-regulation of relative expression of *Ddc* in *TH* mutant in figure 11B. *TH* and *Ddc* are completely different genes, and both encoding enzymes, not coding transcription factors. The authors should explain why *Ddc* expression is down-regulated in *TH* mutants.

Thank you for your reminding. We have explained this in question 8. We have added discussion of the relationship between *TH* and *Ddc* gene interactions to the discussion section of the article, please see page 18, lines 318-328. We have modified it in Table 4.

12. Line 294-295

In figure 10C, the authors showed absorbance at 490 nm. Are the bars in this graph the mean and standard error? The authors should show the sample size (numbers of nymphs) and statistics. What is the meaning of asterisk on the gray bar?

Thank you for your advice. The bars in this graph the mean and standard error. Three 1st-instar nymphs of the same size were used in the experiment. A * indicates a P value less than 0.05, please see page 36, lines 501.

13. Line 297-300, figure 11

In line 297, the authors describe "three cuticle tanning pathway gene: dopamine decarboxylase". I think *Ddc* is a short gene name of "dopa decarboxylase", not "dopamine decarboxylase". And what is the meaning of "three"? Does *Gryllus bimaculatus* have three *Ddc* genes? The authors should add the information about "three" cuticle tanning pathway gene in Introduction or other appropriate sentences.

In figure 11B, the authors showed relative expression of *Ddc* in WT and Mutant. In this figure, did the authors set bar of WT as 1 or not?

The authors should explain the meaning of four asterisks on the gray bar of *Ddc* relative expression in *TH* mutant.

On the vertical bar, the authors labeled as "Gb*DDC*", but it should be "*Ddc*", in consistent with the main text.

Thank you for your reminding.

The word "Three" is a writing error and has been corrected in the article, please see cuticle tanning pathway gene page 37, lines 497-500.

We have performed a new analysis of the relative expression levels of *Ddc* genes, see Fig. 12. In addition, new additions have been made to the description of Fig. 12, see page 37, lines 497-500. In the data analysis, we have normalized the data again. Four stars represents a P value less than 0.0001.

In this article, we have changed "*GbDDC*" to "*Ddc*".

14. Line 316-317

The authors discussed about off-target effects. It is necessary to investigate whether there are off-target effects on the *Ddc* gene in *TH* mutants, at least, in relation to the down-regulation of *Ddc* expression.

Thank you for your advice. We have added discussion of the relationship between *TH* and *Ddc* gene interactions to the discussion section of the article, see page 18, lines 327-338. *TH* genes show different spatio-temporal expression patterns in different species, which may be closely related to the upstream and downstream regulatory elements (Gorman et al., 2007; Yu et al., 2011). In *Tenebrio molitor*, the *Ddc* gene is located downstream of the *TH* gene (Mun et al., 2020).

Gorman M J, An C, Kanost M R. Characterization of tyrosine hydroxylase from *Manduca sexta*. Insect biochemistry and molecular biology, 2007, 37: 1327-1337.

Yu H S, Shen Y H, Yuan G X, et al. Evidence of selection at melanin synthesis pathway loci during silkworm domestication. Molecular biology and evolution, 2011, 28: 1785-1799.

Mun S, Noh M Y, Kramer K J, et al. Gene functions in adult cuticle pigmentation of the yellow mealworm, *Tenebrio molitor*. Insect biochemistry and molecular biology, 2020, 117: 103291.

15. Line 342

The authors described that target site of *TH* is at the fifth exon. In line 95 and figure 1, The authors described target site is on the seventh exon. Which is correct?

Thank you for your reminding. That the target of the *TH* gene on the seventh exon is correct and has been modified in the article, see page 17, line 306.

16. Line 346

The authors discuss about the defective wings and legs, but no photo in figures or description in Result section. The authors should show the abnormal morphologies in the wings and legs.

Thank you for your advice. We have added the phenotype of leg and wing deformities in new Fig. 5.

Minor comments:

Line 95-96

I think the target site of *TH* gene in on the sixth exon. Please confirm the position.

By comparing the sequences of *TH* gene in the transcriptome database, we found that our target was the seventh exon of *TH* gene. We have also identified the sequence and the target by phylogenetic analysis.

Title

"and" should be non-italic in title "Tyrosine Hydroxylase and Yellow genes"

Lines 93-95

GBI_09528-RA is not an accession number of NCBI. The authors cite the reference written by Ylla et al, but the reference is for cricket genome sequence, not for transcriptome database.

Line 126

"sgRNA" in not mRNA.

Table 1

The authors should show the nucleotide sequences of qPCR primers for *Ddc*.

Line 199

"qRT-PCR" should be "qPCR".

Line 200-201

"Student'st-test "should be "Student's t-test".

Line 204

The authors should cite figure 1 in the sentence.

Line 206

"yellow" should be in italic.

Line 235

"dysfunction tyrosine *yellow-y*" would be "dysfunction *yellow-y*"

Line 237 and 240

The authors showed the percentages of mutant phenotype as "71.43%", "58.5%" and "55.6%". Does it make sense to have different decimal places represented? I feel that the decimal places should be aligned.

Line 265 and 266

"larvae" should be "nymphs"

Line 267

"wild egg" should be "wild type eggs"

Line 269

I don't know what "missing gene" means. Does it mean "missing nucleotides" ?

Line 280

The authors showed "*TH*" and "*yellow-y*" in the figure legend, but in figure 9, gene names are shown as ''*GbTH*" and "Gb*yellow-y*" at the vertical axes. These gene names should be "*TH*" and "*yellow-y*".

Line 281

"Aiactin" should be "beta-actin".

Line 288

"1-st instar" should "1st-instar".

Line 313

Add the appropriate references in "mRNA (ref)".

Line 334

"holometabola and hemimetabola" should be "holometabolous and hemimetabolous”.

We thank the reviewer for these suggestions. We have revised them all.
