# Supplementary material for: CRISPR/Cas9-Mediated genomic knock out of tyrosine hydroxylase and yellow genes in cricket Gryllus bimaculatus
Source: PLoS One. 2023 Apr 10;18(4):e0284124. doi: 10.1371/journal.pone.0284124 (PMC10085040; doi:10.1371/journal.pone.0284124)
Supplement: Attachment — Submitted filename: Response to reviewers.docx [file pone.0284124.s011.docx]

**Dear** **Editor,**

We would like to thank you first for all the suggestions of our manuscript (Manuscript Number: PONE-D-22-04580) entitled “CRISPR/Cas9-Mediated Genomic Knock out of Tyrosine Hydroxylase and Yellow genes in Cricket *Gryllus bimaculatus*”. We really appreciate your help and patience. We have seriously thought about the suggestions and provided our response to reviewers.

According to the comments of reviewer 2, we have shifted the focus of the paper to the study on the function of *TH* gene and *yellow-y* gene. We reorganized the abstract and clarified the functions of *TH* gene and *yellow-y* gene. “*Tyrosine hydroxylase* (*TH*) and *yellow-y* are involved in insect melanin and the catecholamine biosynthesis pathway. Compared with the control group, mutations of *TH* and *yellow-y* genes were defective in pigmentation. The result of quantitative real-time PCR analysis revealed that *TH* and *yellow-y* were down-regulated in mutants. Most mutations of the *TH* gene died by the first instar, and the only adult had significant defects in the wings and legs. Therefore, *TH* gene is very important for the growth and development of *G. bimaculatus*,” please see lines 28 to 39 on page 2.

In the introduction, we add the process of melanosis. “The function of *TH* gene and *yellow-y* gene was emphasized. Insect cuticle is soft and pale when it is initial synthesized. The stratum corneum hardening process is complex (Andersen, 2012). During the sclerotization process, tyrosine hydroxylase (TH) is the initial rate-limiting synthetic enzyme involved in biosynthesis of 3,4-dihydroxyphenylalanine (DOPA) (Nagatsu et al., 1964; Axelrod, 1971). DOPA is a significant melanin precursor of quinones which are essential for exoskeleton pigmentation and hardening of the cuticle (Hultmark, 1993; Christensen et al., 2005). Ddc, a pyridoxal-5-phosphate-dependent enzyme, catalyzes the conversion of DOPA to dopamine which is an important neuro-transmitter (Eveleth et al., 1986; Scholnick et al., 1986). Insect major royal jelly proteins (MRJP) or Yellow proteins contain an approximately 300 amino acid-long MRJP domain and were initially identified in the royal jelly proteins that play a central role in honeybee development (Albert et al., 1999).” Please see lines 74 to 83 on page 4.

In the result, we combine the original part 2 and Part 4. So the order of the graph is adjusted according to the content. We changed the original Figure 2 into the supplementary Figure 1. We re-matched the sequence and target of *yellow-y* gene and determined that AGG was PAM sequence, so we re-labeled AGG sequence in Figure 2 and Figure 3. We combined Table 2 and Table 3 and unified the format.

“*TH* participates in the first step of DOPA production from tyrosine (Futahashi and Fujiwara, 2005; Gorman et al., 2007). In *Tenebrio molitor* and *G. bimaculatus*, the *Ddc* gene is located downstream of the *TH* gene (Mun et al., 2020; Seike et al., 2022). Similar phenotypes were obtained from RNAi silencing *TH* gene and *Ddc* gene expression levels in *G. bimaculatus*. In this study we found that the expression level of *Ddc* gene was significantly down-regulated in *TH* mutants of 1st-instar nymphs. The directly interaction of the two genes in *G. bimaculatus* still need further verification.” In the follow-up experiment, we will detect the expression levels of *TH* and *Ddc* genes at different instars. Please see lines 370 to 377 on page 20.

In a recent study, we confirm that the homozygotes of *TH* mutants are lethal, so we could not obtain any homozygotes. We preliminarily concluded that *TH* gene not only affects the pigmentation of *G. bimaculatus*, but is also crucial for the growth and development of *G. bimaculatus*. In future studies, we will study the function of *TH* gene and *yellow-y* gene respectively, and try to construct double knockout mutants of *TH* gene and *yellow-y* gene.

There are the reference of the responses,

Andersen S O. Cuticular sclerotization and tanning[M]//Insect molecular biology and biochemistry. Academic Press, 2012: 167-192.

Nagatsu T, Levitt M, Udenfriend S. Tyrosine hydroxylase: the initial step in norepinephrine biosynthesis. Journal of Biological Chemistry, 1964, 239(9): 2910-2917.

Axelrod J. Noradrenaline: fate and control of its biosynthesis. Science, 1971, 173(3997): 598-606.

Hultmark D. Immune reactions in Drosophila and other insects: a model for innate immunity. Trends in Genetics, 1993, 9(5): 178-183.

Christensen B M, Li J, Chen C C, et al. Melanization immune responses in mosquito vectors. Trends in parasitology, 2005, 21(4): 192-199.

Eveleth D D, Gietz R D, Spencer C A, et al. Sequence and structure of the dopa decarboxylase gene of Drosophila: evidence for novel RNA splicing variants. The EMBO journal, 1986, 5(10): 2663-2672.

Scholnick S B, Bray S J, Morgan B A, et al. CNS and hypoderm regulatory elements of the *Drosophila melanogaster* dopa decarboxylase gene. Science, 1986, 234(4779): 998-1002.

Albert S, Bhattacharya D, Klaudiny J, et al. The family of major royal jelly proteins and its evolution. Journal of Molecular Evolution, 1999, 49(2): 290-297.

Futahashi R, Fujiwara H. Melanin-synthesis enzymes coregulate stage-specific larval cuticular markings in the swallowtail butterfly, *Papilio xuthus*. Development genes and evolution, 2005, 215(10): 519-529.

Gorman M J, An C, Kanost M R. Characterization of tyrosine hydroxylase from *Manduca sexta*. Insect biochemistry and molecular biology, 2007, 37(12): 1327-1337.

Mun S, Noh M Y, Kramer K J, et al. Gene functions in adult cuticle pigmentation of the yellow mealworm, *Tenebrio molitor*. Insect biochemistry and molecular biology, 2020, 117: 103291.

Seike H, Nagata S. Different transcriptional levels of Corazonin, Elevenin, and PDF according to the body color of the two-spotted cricket, *Gryllus bimaculatus*. Bioscience, Biotechnology, and Biochemistry, 2022, 86: 23-30.

Reviewer #1: Although the authors responded well to the reviewers’comments, several minor changes are still required.
In Table 1, Please describe the differences of the nucleotide shown capital and small letters.
Please match the decimal place that used in the main text and table.
In Figure 1, UTRs should be UTR.

We thank the reviewer for these suggestions. We have revised them all.

Reviewer #2: The authors reworked Figures properly and modified Tables with appropriate values. The authors cited new references and added discussion, but the research focus of this manuscript was still to establish and optimize the genome editing strategy using Cas9 protein, and the authors did not shift to the research focus to pigmentation process. The genome editing strategy using Cas9 protein were already established as I mentioned through the review comments, so the novelty of this manuscript is none if the authors insist to publish the manuscript as methods paper. In addition, I recommended to add discussion about the *Ddc* gene expression in previous review comment, but was not enough discussed. I strongly recommend all authors must read and check all the text, figures and tables, because there are lots of careless mistakes in this manuscript.

We thank the reviewer for these very meaningful suggestions.

We have removed the discussion for methods and the conclusions for methods. We have also made a new combination of the experimental results, which are mainly divided into: Identification and analysis of *TH* gene and *yellow-y* gene, The effectiveness of *TH* gene and *yellow-y* gene Targeting, Phenotypic analysis and Suppression of *TH* impairs *G. bimaculatus* pigmentation and Dopamine synthesis.

We reorganized the abstract and clarified the functions of *TH* gene and *yellow-y* gene. “*Tyrosine hydroxylase* (*TH*) and *yellow-y* are involved in insect melanin and the catecholamine biosynthesis pathway. Compared with the control group, mutations of *TH* and *yellow-y* genes were defective in pigmentation. The result of quantitative real-time PCR analysis revealed that *TH* and *yellow-y* were down-regulated in mutants. Most mutations of the *TH* gene died by the first instar, and the only adult had significant defects in the wings and legs. Therefore, *TH* gene is very important for the growth and development of *G. bimaculatus*,” please see lines 28 to 39 on page 2.

In the introduction, we add the process of melanosis. “The function of *TH* gene and *yellow-y* gene was emphasized. Insect cuticle is soft and pale when it is initial synthesized. The stratum corneum hardening process is complex (Andersen, 2012). During the sclerotization process, tyrosine hydroxylase (TH) is the initial rate-limiting synthetic enzyme involved in biosynthesis of 3,4-dihydroxyphenylalanine (DOPA) (Nagatsu et al., 1964; Axelrod, 1971). DOPA is a significant melanin precursor of quinones which are essential for exoskeleton pigmentation and hardening of the cuticle (Hultmark, 1993; Christensen et al., 2005). Ddc, a pyridoxal-5-phosphate-dependent enzyme, catalyzes the conversion of DOPA to dopamine which is an important neuro-transmitter (Eveleth et al., 1986; Scholnick et al., 1986). Insect major royal jelly proteins (MRJP) or Yellow proteins contain an approximately 300 amino acid-long MRJP domain and were initially identified in the royal jelly proteins that play a central role in honeybee development (Albert et al., 1999).” Please see lines 74 to 83 on page 4.

In the result, we combine the original part 2 and Part 4. So the order of the graph is adjusted according to the content. We changed the original Figure 2 into the supplementary Figure 1. We re-matched the sequence and target of *yellow-y* gene and determined that AGG was PAM sequence, so we re-labeled AGG sequence in Figure 2 and Figure 3. We combined Table 2 and Table 3 and unified the format.

“*TH* participates in the first step of DOPA production from tyrosine (Futahashi and Fujiwara, 2005; Gorman et al., 2007). In *Tenebrio molitor* and *G. bimaculatus*, the *Ddc* gene is located downstream of the *TH* gene (Mun et al., 2020; Seike et al., 2022). Similar phenotypes were obtained from RNAi silencing *TH* gene and *Ddc* gene expression levels in *G. bimaculatus*. In this study we found that the expression level of *Ddc* gene was significantly down-regulated in *TH* mutants of 1st-instar nymphs. The directly interaction of the two genes in *G. bimaculatus* still need further verification.” In the follow-up experiment, we will detect the expression levels of *TH* and *Ddc* genes at different instars. Please see lines 370 to 377 on page 20.

In a recent study, we confirm that the homozygotes of *TH* mutants are lethal, so we could not obtain any homozygotes. We preliminarily concluded that *TH* gene not only affects the pigmentation of *G. bimaculatus*, but is also crucial for the growth and development of *G. bimaculatus*. In future studies, we will study the function of *TH* gene and *yellow-y* gene respectively, and try to construct double knockout mutants of *TH* gene and *yellow-y* gene.

There are the reference of the responses,

Andersen S O. Cuticular sclerotization and tanning[M]//Insect molecular biology and biochemistry. Academic Press, 2012: 167-192.

Nagatsu T, Levitt M, Udenfriend S. Tyrosine hydroxylase: the initial step in norepinephrine biosynthesis. Journal of Biological Chemistry, 1964, 239(9): 2910-2917.

Axelrod J. Noradrenaline: fate and control of its biosynthesis. Science, 1971, 173(3997): 598-606.

Hultmark D. Immune reactions in Drosophila and other insects: a model for innate immunity. Trends in Genetics, 1993, 9(5): 178-183.

Christensen B M, Li J, Chen C C, et al. Melanization immune responses in mosquito vectors. Trends in parasitology, 2005, 21(4): 192-199.

Eveleth D D, Gietz R D, Spencer C A, et al. Sequence and structure of the dopa decarboxylase gene of Drosophila: evidence for novel RNA splicing variants. The EMBO journal, 1986, 5(10): 2663-2672.

Scholnick S B, Bray S J, Morgan B A, et al. CNS and hypoderm regulatory elements of the *Drosophila melanogaster* dopa decarboxylase gene. Science, 1986, 234(4779): 998-1002.

Albert S, Bhattacharya D, Klaudiny J, et al. The family of major royal jelly proteins and its evolution. Journal of Molecular Evolution, 1999, 49(2): 290-297.

Futahashi R, Fujiwara H. Melanin-synthesis enzymes coregulate stage-specific larval cuticular markings in the swallowtail butterfly, *Papilio xuthus*. Development genes and evolution, 2005, 215(10): 519-529.

Gorman M J, An C, Kanost M R. Characterization of tyrosine hydroxylase from *Manduca sexta*. Insect biochemistry and molecular biology, 2007, 37(12): 1327-1337.

Mun S, Noh M Y, Kramer K J, et al. Gene functions in adult cuticle pigmentation of the yellow mealworm, *Tenebrio molitor*. Insect biochemistry and molecular biology, 2020, 117: 103291.

Seike H, Nagata S. Different transcriptional levels of Corazonin, Elevenin, and PDF according to the body color of the two-spotted cricket, *Gryllus bimaculatus*. Bioscience, Biotechnology, and Biochemistry, 2022, 86: 23-30.

Major comments:
 
1. The authors modified figure 3 and figure 9. In these figures, the authors showed that GGC sequences just adjacent to 5' side of sgRNA in *yellow-y* gene are the PAM sequences, but could be incorrect. PAM sequences must be NGG for Cas9 and locate just adjacent to 3' side of sgRNA, thus, I think that AGG sequences shown in figure 9D could be the PAM sequences. In figure 3D, I was confused that double peaks were appeared far from PAM sequences, as I pointed out in previous review comments. If the AGG was correct PAM sequences, double peaks in figure 3D were very close to PAM sequences and that is reasonable. The authors have to confirm the correct PAM sequences and should rewrite results and discussion and rework many figures, based on the position of PAM sequences.

We re-matched the sequence and target of *yellow-y* gene and determined that AGG was PAM sequence, so we re-labeled AGG sequence in Figure 2 and Figure 3.

2. To answer my previous comment, the authors discuss about the decreased expression of *Ddc* in *TH* knock out crickets in line 332-333, citing the genetic loci of *Ddc* and *TH* in *Tenebrio molitor*. However, in *Gryllus bimaculatu*s, *TH* and *Ddc* are found in different scaffolds, thus the situation is completely different to the *Tenebrio molitor*. The authors should show the evidence why *Ddc* was decreased in *TH* knock out crickets or add the appropriate discussion.

*TH* participates in the first step of DOPA production from tyrosine (Futahashi and Fujiwara, 2005; Gorman et al., 2007). In *Tenebrio molitor* and *G. bimaculatus*, the *Ddc* gene is located downstream of the *TH* gene (Mun et al., 2020; Seike et al., 2022). Similar phenotypes were obtained from RNAi silencing *TH* gene and *Ddc* gene expression levels in *G. bimaculatus*. In this study we found that the expression level of *Ddc* gene was significantly down-regulated in *TH* mutants of 1st-instar nymphs. The directly interaction of the two genes in *G. bimaculatus* still need further verification. In the follow-up experiment, we will detect the expression levels of *TH* and *Ddc* genes at different instars. Please see lines 370 to 377 on page 20.

Futahashi R, Fujiwara H. Melanin-synthesis enzymes coregulate stage-specific larval cuticular markings in the swallowtail butterfly, *Papilio xuthus*. Development genes and evolution, 2005, 215(10): 519-529.

Gorman M J, An C, Kanost M R. Characterization of tyrosine hydroxylase from Manduca sexta. Insect biochemistry and molecular biology, 2007, 37(12): 1327-1337.

Mun S, Noh M Y, Kramer K J, et al. Gene functions in adult cuticle pigmentation of the yellow mealworm, *Tenebrio molitor*. Insect biochemistry and molecular biology, 2020, 117: 103291.

Seike H, Nagata S. Different transcriptional levels of Corazonin, Elevenin, and PDF according to the body color of the two-spotted cricket, *Gryllus bimaculatus*. Bioscience, Biotechnology, and Biochemistry, 2022, 86: 23-30.

Minor points
 
Line 5
"knoc kout" should be "knock out" in the short title .
 
Materials and Methods
Some values are described with hyphen and others are without. Authors should unify their description without hyphens.
 
Line 202
Authors explained about gene structure of *TH*, but did not about *yellow-y*, although the figure 1 contains both *TH* and *yellow-y*.
 
Line 237
Fig. 7 should be Fig. 8.
 
Line 248
wild-type should be WT.
 
Line 263-264
Fig. 11A should be Fig. 11A,B and Fig. 11B should be Fig. 11C.
 
Line 480
larval instar should be nymphal instar.
 
Line 495-496
*yellow-y* should be in italic.

We thank the reviewer for these suggestions. We have revised them all.
