# Supplementary material for: CRISPR/Cas9-Mediated genomic knock out of tyrosine hydroxylase and yellow genes in cricket Gryllus bimaculatus
Source: PLoS One. 2023 Apr 10;18(4):e0284124. doi: 10.1371/journal.pone.0284124 (PMC10085040; doi:10.1371/journal.pone.0284124)
Supplement: Attachment — Submitted filename: Response to reviewers(1).docx [file pone.0284124.s012.docx]

**Dear** **Editor,**

We would like to thank you first for all the suggestions of our manuscript (Manuscript Number: PONE-D-22-04580) entitled “CRISPR/Cas9-Mediated Genomic Knock out of Tyrosine Hydroxylase and Yellow genes in Cricket *Gryllus bimaculatus*”. We really appreciate your help and patience.

We have seriously thought about the suggestions and provided our response to reviewers.

It comes to our notice that some sentences are significantly or even completely identical to those of other publications. Examples are:

Lines 61 - 63

Lines 71 - 79

Lines 107 - 108

Lines 112 - 115

Lines 121 - 122

Lines 124 - 127

Lines 128 - 129

Lines 159 - 161

Lines 181 - 183

Lines 184 - 193

Lines 264 - 268

Lines 277 - 290

Lines 291 - 293

Lines 314 - 315

Lines 321 - 322

We thank the reviewer for these suggestions. In the writing process of this article, we mainly refer to the work of students in **our** research group (Yang, Y., Wang, Y. H., Chen, X. E., Tian, D., Xu, X., **Li**, **K**., Huang, Y. P. & He, L. (2018). CRISPR/Cas9‐mediated Tyrosine hydroxylase knockout resulting in larval lethality in *Agrotis ipsilon*. Insect science, 25(6), 1017-1024.). The experimental method is similar to the related research topic in this article. Therefore, some sentences are similar. In this new version, we have rewritten them all.

Major comment
I pointed out the mistakes of the PAM sequences of yellow-y in my previous comment, and the authors rework the figure 3. However, the PAM sequences of yellow-y indicated in Supplementary Fig 2 were still incorrect. The authors should rework them.
Minor comments
line 205-206:The authors should insert space before the "bp".
line 236 "The legs and wings of the adult is" should be "The legs and wings of the adult are".

As for the issue in Supplementary Fig 2 raised by reviewer 2, we have revised it, please see Supplementary Fig 2.

We thank the reviewer for these suggestions. We have revised them all.
